# Bacterial community diversity and its potential contributions to the flavor components of traditional smoked horsemeat sausage in Xinjiang, China

**DOI:** 10.3389/fmicb.2022.942932

**Published:** 2022-07-27

**Authors:** Lei Jiang, Yu Chen, Li Deng, Fei Liu, Tengbin Wang, Xuewei Shi, Bin Wang

**Affiliations:** ^1^College of Life and Geographical Sciences, Kashi University, Kashi, China; ^2^Food College, Shihezi University, Shihezi, China; ^3^College of Enology, Northwest A&F University, Yangling, China; ^4^Xinjiang Academy of Analysis and Testing, Wulumuqi, China

**Keywords:** smoked horsemeat sausages, microbial community, volatile compounds, physicochemical characteristic, correlation

## Abstract

Smoked horsemeat sausage is a famous fermented traditional food in Xinjiang, China. However, the microbial diversity and its potential contributions to the flavor components of smoked horsemeat sausage are unclear. In this study, the microbial community and flavor components of smoked horsemeat sausage from six regions of Xinjiang were measured by using amplicon sequencing and headspace solid-phase microextraction combined with gas chromatography–mass spectrometry (HS-SPME-GC–MS) technology, respectively. Relations among microbial communities, flavor components and environmental factors were subsequently predicted based on redundancy analysis (RDA) and Monte Carlo permutation tests. Although smoked horsemeat sausage samples from different regions possessed distinct microbial communities, lactic acid bacteria (LAB) were identified as the dominant consortium in smoked horsemeat sausage. *Lactobacillus*, *Vagococcus*, *Lactococcus*, and *Carnobacterium* were detected at high abundance in different sausages. The moisture content, nitrite content, and pH of the sausage might be important factors influencing the dominant bacterial community, according to the RDA. Among the dominant consortia, the eight core bacterial genera showed considerable correlations with the formation of sixteen volatile compounds in smoked horsemeat sausage based on multivariate statistical analysis. For example, the levels of *Leuconostoc* and *Lactobacillus* were positively correlated with those of 1-hexadecanol, hexyl acetate, 2-methyl-phenol, 1-pentanol, d-limonene, and 2-heptanone, and the levels of *Leuconostoc*, *Lactobacillus*, and *Weissella* were negatively correlated with those of 1-octanol, acetic acid, octanal, heptanal, and 1-hexanol. This study will provide a theoretical basis for understanding the microbial metabolic modes of Xinjiang smoked horsemeat sausages.

## Introduction

Meat processing, such as fermentation, supplemented with sodium chloride treatment and a drying process, is a key method for extending the shelf life of the raw materials and increasing their economic value (Dias et al., 2020). In China, Xinjiang is one of the most important regions for the production of processed meat (Zhang et al., 2019). Kazakh smoked horsemeat sausages, a traditional fermented meat product, are very popular in Xinjiang and are mostly prepared with traditional techniques (Hu et al., 2020). This product stands out for its unique organoleptic characteristics. Compared with traditional fermented sausages from other parts of China, smoked horsemeat sausages use horse meat instead of pork because horse meat has approximately 20% less fat and cholesterol content than pork and is more beneficial to human health (Lorenzo et al., 2014). Generally, this product is prepared between November and December. First, the horse ribs are cut into strips and mixed with salt, pepper, sugar and other condiments together with the ribs, and then stuffed into natural horse intestine casings. The meat is then placed on the wooden frame of a soil block wall, smoked with pine branch smoke, stored in an open room for 30–40 days for air drying and natural fermentation, and finally harvested to obtain mature smoked horsemeat sausage (Lu et al., 2015). Usually, in the sausage preparation process, meat cutting, enema, and smoking are performed with exposure to an open environment, and many microorganisms from the raw materials and the environment participate in these processes (Juarez-Castelan et al., 2019; Liu et al., 2019; Zhang et al., 2019). The complex microbial ecosystem is formed, which plays a vital role in the quality and sensory characteristics of the sausages.

The distinct characteristics of fermented food from different geographical regions are essential in defining the economic and cultural value of luxury meat products (Dias et al., 2020; Gao et al., 2021). Kazakh smoked horsemeat sausages are similarly regarded as a kind of microbiologically stable fermented products (Zhao et al., 2018). Ecological studies of these fermented meat products are of primary importance to understand the physical and chemical changes and to characterize their distinct sensory profile. A variety of microbes can exist in the raw materials used to produce sausages, which results in the diversity of the microbial community in sausages and may lead to inconsistent quality of the sausages. With the development of 16S rRNA sequencing technology and flavor evaluation, the bacterial community structure and quality in meats have been widely and successfully determined. As expected, lactic acid bacteria (LAB) are always the dominant flora and are responsible for the fermentation of sausages, but different species have been reported to be involved (Chen et al., 2017). The primary bacteria in Chinese Sichuan fermented sausages were *Lactobacillus* spp., *Weissella* spp., and *Pediococcus* spp. (Wang et al., 2019). Chen et al. (2019) also found that core microbes and sensory characteristics of all examined sausages were affected by the NaCl and moisture levels. However, the microbial diversity as well as the functions of different microbes involved in fermentation in fermented meat production remain to be fully characterized. Furthermore, there is a growing body of significant observational and research providing evidence of the relationship between volatile-compound profiles and the core microbiota in fermented process (Chen et al., 2020; Hu et al., 2020; Zheng et al., 2021). It is reported that esters showed the highest coefficient of variation among five Spanish dry fermented sausage, followed by phenols and terpenes (Ansorena et al., 2001). Hu et al. (2020) detected a total of 120 volatile compounds, mainly including alcohols, acids, aldehydes, ketones, esters, and terpenes, in sausages from five different regions, and explained the potential correlations between the core bacteria and 64 major volatile compounds. Meanwhile, Chen et al. (2019) also found there were significantly discrepant in color, saltiness aroma, acid taste, texture and acceptability among fermented sausage with different treatments. Therefore, it has become especially important to understand the complex correlations between the core bacteria and characteristic flavors of fermented meat products.

In this study, the high-throughput sequencing (HTS) approach was used to determine the bacterial diversity, and volatile compounds were detected via headspace solid-phase microextraction combined with gas chromatography–mass spectrometry (HS-SPME-GC–MS). In addition, physical characteristics of smoked horsemeat sausages from different regions in Xinjiang, China, including the moisture content, water activity (aw), pH, nitrite content and total lactic acid content, were examined. Ultimately, the relationship between volatile compounds and microbial communities was predicted by correlation analysis. The results of this study may be helpful for understanding the role of indigenous microorganisms in the formation of volatile aromas and standardizing the industrial production of Kazakh smoked horsemeat sausages.

## Materials and methods

### Samples collection

The traditional smoked horsemeat sausages used were manufactured in the main production area of six Kazakh herdsman settlement states in Xinjiang, China, including Yili (YL), Mulei (ML), Aletai (AL), Bole (BZ), Balikun (BL), and Tacheng (TC). At least three families were selected from each production area, and 2.0 kg of sausage was collected from each family. First, the horse lean meat and back fat are cut into strips and mixed with 2–2.5% salt and 1.5–2% sugar, Then smoked with pine branch smoke and stored in an open room for 30–40 days for air drying and natural fermentation. These sausages were matched in terms of process and formulation. At the end of fermentation, the sausages were transported to the laboratory in a sterilized ice box and stored at appropriately −80°C for further analysis.

### Moisture content, water activity, pH, nitrite content, and total lactic acid content

The moisture content was measured according to the AOAC procedures (AOAC, 1995). The aw was determined by an AquaLab Pawkit (Decagon Devices, Inc., Pullman, WA, United States). The pH was measured exactly as described by Yu et al. (2021). Measurement of the nitrite and total lactic acid levels was performed with a Lactic Acid (LA) Content Detection Kit (Beijing Solarbio Science & Technology Co., Ltd., Beijing, China) and Nitrite Content Detection Kit in Food (Beijing Solarbio Science & Technology Co., Ltd., Beijing, China), respectively, according to the manufacturer’s instructions.

### DNA extraction, amplicon sequencing, and diversity analysis

Total genomic DNA from meat samples (0.2 g) was extracted using the cetyl trimethyl ammonium bromide/sodium dodecyl sulfate (CTAB/SDS) method and DNA quality was monitored using 1% (w/v) agarose gel electrophoresis (Brandfass and Karlovsky, 2008).

From the DNA templates, the V3-V4 variable regions of the 16S rRNA gene were amplified using the specific primers 338F (5′-ACTCCTACGGGAGGCAGCAG-3′) and 806R (5′-GGACTACHVGGGTWTCTAAT-3′) (Wang et al., 2018). The following PCR amplified conditions were used: initial denaturation (96°C, 3 min), 30 cycles of denaturation (95°C, 30 s), annealing (60°C, 45 s), extension (72°C, 45 s), and a final extension (72°C for 10 min). The resulted PCR products were extracted from a 2% (w/v) agarose gel and further purified using the AxyPrep DNA Gel Extraction Kit (Axygen Biosciences, Union City, CA, United States). Sequencing libraries were then generated using a NEXTFLEX Rapid DNA-Seq Kit (Bioo Scientific, PA, United States). Finally, the purified PCR products was sequenced with a Illumina MiSeq PE300 platform (Illumina, San Diego, CA, United States) at Microeco Tech Co., Ltd. (Shenzhen, China).

High-throughput sequencing raw data were initially processed through the QIIME2. The QIIME2 feature-classifier plugin was then used to align ASV sequences to a pre-trained GREENGENES 13_8 97% database (trimmed to the V3-V4 region bound by the 338F/806R primer pair) to generate the OUT taxonomy table. Diversity metrics were calculated using the core-diversity plugin within QIIME2. Feature level alpha diversity indices, such as observed OTUs, Chao1 richness estimator, Shannon diversity index, and Faith’s phylogenetics diversity index were calculated to estimate the microbial diversity within an individual sample (Gao et al., 2019; Chen et al., 2020).

### Volatile compound analysis

As described by Hu et al. (2020) and Franciosa et al. (2021), volatile compounds in smoked horsemeat sausages were extracted by HS-SPME and analyzed using a GC/MS system (Pegasus GC-HRT 4D Plus, LECO, PA, United States) with some modifications. Volatile compounds sampling was performed using 75-μm carboxen/polydimethylsiloxane (CAR/PDMS) (Supelco, PA, United States) solid-phase microextraction (SPME) fiber. The column applied was a DB-Wax column (30 m × 0.25 mm × 0.25 μm). Volatile compounds were extracted from each sausage sample (3.0 g) in a 20 mL vial at 60°C for 50 min under agitation at 250 rpm. Helium (99.999%) at a constant rate of 1 mL/min was used as carrier gas. The oven temperature increased from 40°C for 3 min, again increased at a rate 10°C/min until 230°C, and finally held 230°C for 6 min. MS detector operated at 200°C in full scan mode with total ion current of 70 eV. Volatile compounds were identified by matching the component peak with the standard compound in the NIST 8.0 library and by calculating the retention index relative to a series of standard alkanes (C6–C26) to calculate the linear retention index (LRI) (the degree of similarity was more than 90%). The volatile compounds were quantified by dividing the peak areas of the compounds by the total peak area (expressed as%).

### Bacterial function prediction

Bacterial functional features were identified by Phylogenetic Investigation of Communities by Reconstruction of Unobserved States 2 (PICRUSt2). The bacterial OTUs exported from QIIME2 in standard format are imported into PICRUSt2. The PICRUSt software package is available from https://anaconda.org/bioconda/picrust2 (Langille et al., 2013). Functional enrichment analysis of metabolic pathways was carried out in R 3.3.3 software using the ClusterProfiler package, and the path view package was used for metabolic pathway integration and visualization (Shi et al., 2020; Yao et al., 2020).

### Statistical analysis

Three independent batches of smoked horsemeat sausage (replicates) were collected, and all measurements for each batch of smoked horsemeat sausage were performed three times (three observations). The results are expressed as the mean ± standard error (SE). Significant difference analysis was performed using IBM SPSS statistical software (version 22.0). Spearman correlation analysis was used to estimate relationships between bacterial species using the pheatmap package in R software (version 3.3.3). Spearman correlation analysis of bacteria and volatile compounds was performed using the ggcor package in R software to explore the correlation (He and Chung, 2020; Gao et al., 2021). Principal component analysis (PCA) of bacterial communities and volatile compounds and VIP analysis were performed in SIMCA software (version 14.1). Spearman correlation coefficients between microorganisms and volatile components were calculated by O2PLS and visualized by Cytoscape software (version 3.6.1).

## Results and discussion

### Analysis of physicochemical characteristics in the different sausages

The variations in the physical parameters among smoked horsemeat sausage samples from six different regions are shown in Table 1. The moisture content and aw were evidently separated among different regions in each batch. They ranged in value from 11.57 ± 0.02% to 17.40 ± 0.01% and 0.869 ± 0.002% to 0.965 ± 0.001%, respectively. The moisture content and aw in the TC, YL, and ML samples were higher than those in the AL, BL, and BZ samples (*P* < 0.05). This low water activity in the BZ sample has the benefit of inhibiting the growth of food-borne pathogenic and spoilage bacteria (Wang et al., 2018). In addition, to prevent sausage contamination, nitrite is commonly used a preservative and added to the sausage. Although sodium nitrite can improve the color and inhibit the spoilage of fermented sausages, carcinogenic nitrosamines further formed by sodium nitrite pose a threat to human health (Kim et al., 2017; Zhu et al., 2020). In our samples, the concentrations of nitrite were far below Chinese national standard GB2762-2017 (0.435 μmol/g). The nitrite residues in the samples from the six regions ranged in concentration from 0.0790 μmol/g (TC) to 0.1168 μmol/g (YL). The differences were attributed to the variation in raw materials and production techniques. The pH values were significantly different among the six samples (*P* < 0.05). The pH value of the sausages fluctuated between 5.39 (TC) and 5.82 (BZ); however, the pH values of the samples from AL and BL were 4.44 and 4.54, respectively. The results indicated that the samples from AL and BL were more acidic than the others. The variance in the pH value might result in the utilization of carbohydrates and subsequent production of organic acids, such as acetic acid and lactic acid, by bacteria (mainly LAB) (Özyurt et al., 2016; Juarez-Castelan et al., 2019). Interestingly, the total lactic acid content of the six samples was not consistent with the change in pH values. The total lactic acid levels in the TC and YL samples were 0.3448 ± 0.0193 μmol/g and 0.6259 ± 0.0300 μmol/g, respectively, while the levels in the other samples ranged from 1.2951 ± 0.0558 μmol/g to 1.7520 ± 0.0751 μmol/g.

**TABLE 1:** Physical properties of smoked horse meat sausages from different regions.

Sample	Moisture content (%)	aw	Nitrite (μ mol/g)	pH	Total lactic acid (μ mol/g)
TC	16.25 ± 0.01a	0.949 ± 0.001a	0.079 ± 0.016a	5.39 ± 0.02a	0.345 ± 0.020a
AL	11.57 ± 0.02b	0.927 ± 0.003b	0.099 ± 0.002b	4.44 ± 0.07c	1.660 ± 0.099d
BL	14.34 ± 0.02ab	0.933 ± 0.001b	0.089 ± 0.002bc	4.54 ± 0.04c	1.752 ± 0.075d
BZ	13.81 ± 0.01ab	0.869 ± 0.002c	0.0831 ± 0.017c	5.82 ± 0.06b	1.295 ± 0.056c
YL	17.21 ± 0.01a	0.965 ± 0.001a	0.117 ± 0.002d	5.47 ± 0.09a	0.626 ± 0.030b
ML	17.40 ± 0.01a	0.957 ± 0.001a	0.093 ± 0.001b	5.74 ± 0.01b	1.312 ± 0.067c

Mean value ± SE (n = 3) followed by different lower-case letters in each column indicate significant differences at P < 0.05.

### Bacterial richness and diversity

**FIGURE 1:**
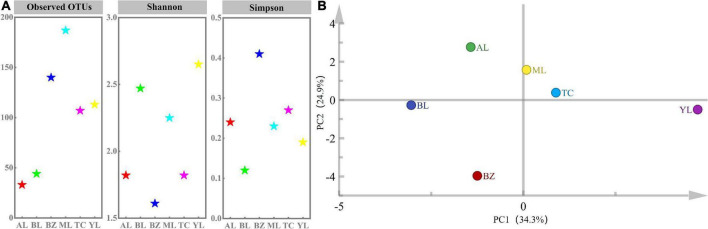
Alpha diversity **(A)** and principal component analysis (PCA) **(B)** of smoked horsemeat sausages from different regions of Xinjiang based on bacterial OTUs.

A data set containing an average of 32,902 raw reads (range: 22,174–38,618) and a total of 561,608 clean reads based on 16S rRNA gene sequences was generated using MiSeq. All the sequences were clustered at a threshold value of 97%, with a total of 1,721 OTUs in all samples (range: 33–187) (Supplementary Table 1). These OTUs were clustered into 21 phyla, 57 classes, 106 orders, 159 families, and 359 genera. The values of the Shannon indexes in YL and BL were higher than those other four samples, BZ had the highest values of Simpson, ML had the highest values of ACE and Chao 1, followed by BZ, YL, TC, BL, and AL, which indicated the richness of the bacteria among the six samples (Figure 1; Supplementary Table 1). It has been reported that raw materials directly affect the microbial diversity of fermented sausages (Hu et al., 2020). The complex material composition of horsemeat, including proteins, natural carotenoids, calcium, and chitin, might be responsible for the different alpha diversity index values of the six regional samples. The Good’s coverage (a sampling completeness indicator) values of all the regional smoked horsemeat sausages were over 99%, suggesting that sufficient bacterial diversity was obtained by the sampling regimen used. In addition, rarefaction analysis showed an approximation to an asymptote (Supplementary Figure 1), suggesting that the sequences were representative of the bacterial diversity in the six samples from different regions.

The distribution of OTUs among the different samples was evaluated by a petal diagram. As shown in Supplementary Figure 2, 33, 113, 107, 187, 140, and 44 OTUs were identified in the AL, YL, TC, ML, BZ, and BL samples, respectively. Overall, seven OTUs were shared by all samples, which revealed a low level of similarity in bacterial diversity among the samples. PCA was applied to establish a model and revealed intimate connections among different smoked horsemeat sausages, in which principal component 1 (PC1) and PC2 explained 34.3 and 24.9% of the variance for bacteria (Figure 1B), respectively. In fact, almost all of the samples were separated in the PCA plot, but the AL and BL and ML and TC samples were closer to each other than to the other samples. This result suggested that a specific bacterial community was present in each sample and that the microbiota was strongly affected by regional factors.

### Bacterial composition and community differences in the different sausages

**FIGURE 2:**
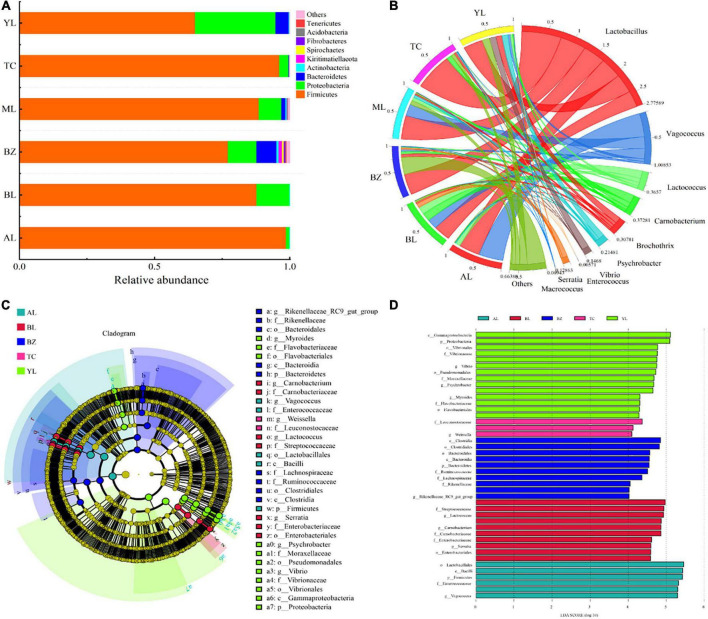
Analysis of bacterial community composition and difference in different smoked horsemeat sausages. Relative abundance of bacteria at the phylum **(A)** and genus **(B)** levels. **(C)** Taxonomic representation of statistically and biologically consistent differences among different smoked horsemeat sausages. Colored clades represent significant differences with an LDA threshold of 4.0. **(D)** Histogram of the LDA scores for differentially abundant taxa.

The relative abundance of different bacterial microorganisms in all sausage samples is shown in Figure 2A (phylum level) and Figure 2B (genus level). Nine bacterial phyla (*Firmicutes*, *Proteobacteria*, *Bacteroidetes*, *Actinobacteria*, *Kiritimatiellaeota*, *Spirochaetes*, *Fibrobacteres*, *Acidobacteria*, and *Tenericutes*) were detected, but more than 95% of the annotated reads were assigned to *Firmicutes* and *Proteobacteria*. Specifically, in the AL and BL samples, the overall proportion of *Firmicutes* and *Proteobacteria* reached 100%. Almost all of the identified phyla were contained in the BZ samples, which is consistent with results obtained from the evaluation of alpha diversity.

For a more detailed analysis of the bacterial dynamics associated with Xinjiang smoked horsemeat sausages in different regions, bacterial compositions at the genus level were determined (Figure 2B). A total of 359 genera were shared by all the sausage samples, and the top ten genera, namely, *Lactobacillus* (32.52–75.10%), *Vagococcus* (4.36–47.00%, except the TC sample), *Lactococcus* (0.05–15.27%), *Carnobacterium* (1.11–16.15%), *Brochothrix* (0.1–11.97%), *Psychrobacter* (1.10–11.08%), *Vibrio* (0.01–13.11%), *Serratia* (0–8.48%), *Enterococcus* (0–0.18%), and *Macrococcus* (0–0.26%), were detected at high abundance in different sausages (Figure 2B). LAB (*Lactobacillus* and *Lactococcus*) and *Vagococcus* (average of 16.81%) prevailed among all production regions of smoked horsemeat sausages. The resulting ripening properties of the sausages were strongly attributed to the microflora because fermentation is dominated by microorganisms (Ammor and Mayo, 2007; Tabanelli et al., 2012). Owing to their acidification and enzymatic (protease and lipase) activities, LAB are regarded as the main groups of bacteria with technological significance during fermentation and ripening of fermented meat products (Aro et al., 2010; Wang et al., 2018). LAB are capable of growing at lower pH and aw values, and acidification by LAB alters the quality and aroma of the final product to varying degrees (Leroy et al., 2010; Ravyts et al., 2010). In our study, LAB, accounting for 52.36% on average as the predominant bacteria, were detected in all the sausages. The results are consistent with previous studies reported by Hu et al. (2020).

To explore the bacterial OTUs that most likely contributed to the significant differences in bacterial community compositions among the six sausage samples, biomarker analysis was performed by using the linear discriminant analysis (LDA) effect size (LEfSe) method. At the genus level, *Serratia* was identified as an indicator for the Balikun region (BL) (Figures 2C,D). *Vibrio*, *Psychrobacter*, and *Myroides* had significantly higher relative abundances in the Yili region (YL), and *Rikenellaceae_RC9_gut_group* contributed most to the difference in the Bole region (BZ). Interestingly, characteristic biomarkers in the Mulei region (ML) have not been identified in general. Moreover, 43 bacterial clades showed significant differences with an LDA threshold of 4.0 (Figure 2D). Although the abundant LAB genus *Lactobacillus* was not specifically enriched in all the sausage samples, species of other LAB genera (such as *Lactococcus*) may have the potential to serve as regional biomarkers. However, more evidence is needed for this, such as data from quantitative PCR based on primers designed specifically for these microbes.

### Multivariate analysis and gene function of the bacterial community in the different sausages

**FIGURE 3:**
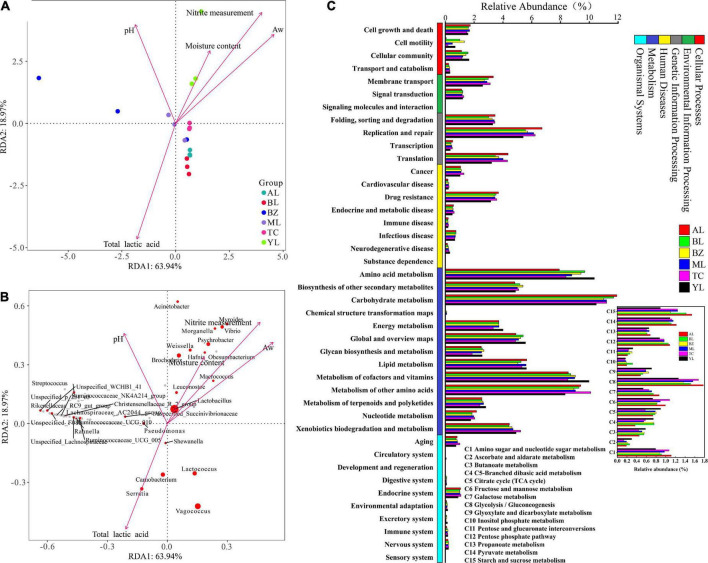
Redundancy analysis (RDA) of physical factors and samples based on the bacterial community **(A,B)** and the abundance of KEGG pathways **(C)** in different smoked horsemeat sausages. In RDA, an acute angle indicates a positive correlation while an obtuse angle indicates a negative correlation between the species and environmental factors. **(B)** A red circle represents a significant bacterial genus, while the circle space represents the abundance of the species. **(C)** Bacterial functions at levels 1 and 2 and carbohydrate metabolism at level 3 are shown.

It is widely believed that environmental factors are probably responsible for the microbial community changes that occur in mixed-microbial consortia (Jung et al., 2014). Redundancy analysis (RDA) was applied to further analyze the relationship between the top 25 species and physical and chemical factors. In the RDA, aw, and especially the moisture content and nitrite were positively correlated in sausages samples from the Yili region (Figure 3A). The YL and ML sausage samples showed positive correlation of pH values. The *Weissella*, *Brochothrix*, *Myroides*, *Psychrobacter*, and *Vibrio* abundances showed correlation in the YL sausage and were greatly affected by moisture content and nitrite, while the total lactic acid content had a significant and positive correlation with *Serratia*, *Lactococcus*, *Vagococcus*, *Carnobacterium*, and *Shewanella* (Figure 3B). Interestingly, *Lactobacillus* had a faintly negative correlation with the total lactic acid content. The changes in *Lactobacillus* abundance might have been caused by the interactions of the species.

The bacterial functional genes predicted by PICRUSt2 in Xinjiang smoked horsemeat sausages were classified into the categories cellular processes, environmental information processing, genetic information processing, human diseases, metabolism, organismal systems, and unclassified (not shown) at level 1 (Figure 3C). A total of 377 KEGG pathways were found, and most of them were classified into metabolism (approximately 41.64%). The pathways belonging to metabolism in level 2 mainly included amino acid metabolism, carbohydrate metabolism, and metabolism of cofactors and vitamins (Figure 3C). The concentration of protein in horsemeat is abundant and reach to 22.5% (Ruiz-Valdepeñas Montiel et al., 2017). The abundant genes about amino acid metabolism seem to be helpful for the development of fermented sausage in our results. The different bacterial genera related to lipid metabolism could make up an important component of the volatile composition in smoked horsemeat sausages (Wang et al., 2021). These tendencies make sense in fermentation process of smoked horsemeat sausages. Among the pathways of carbohydrate metabolism, amino sugar and nucleotide sugar metabolism (C1), fructose and mannose metabolism (C6), glycolysis/gluconeogenesis (C8), the pentose phosphate pathway (C12), pyruvate metabolism (C14), and starch and sucrose metabolism (C15) were detected with high abundance (≥1.0% in at least one sample). The pathways C6, C8, and C14 are all related to the production of lactic acid. Based on the above results, we observed that the region affecting the diversity of microorganisms of smoked horsemeat sausages, could resulted the difference of functions, which might influence the organoleptic profile of the final product.

### Co-occurrence analysis of microbes

**FIGURE 4:**
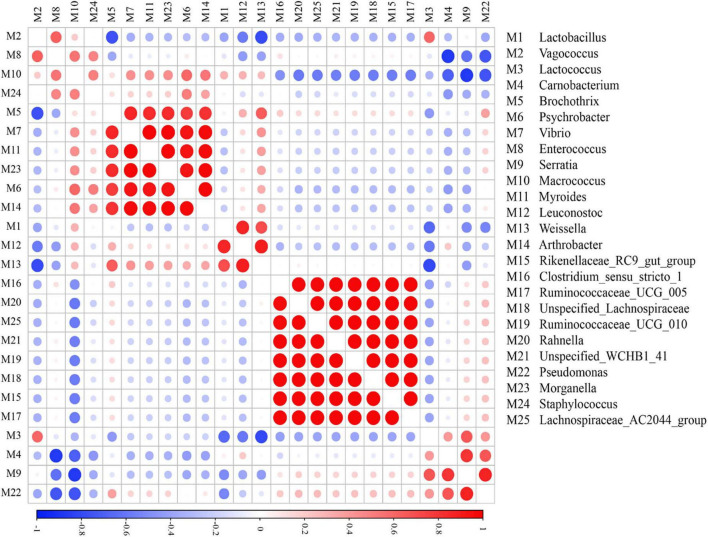
Co-occurrence and exclusion correlations among bacteria at the genus level. Strong correlations are shown by large circles, while weak correlations are shown by small circles.

Microbial co-occurrence networks play a vital role in clarifying the symbiotic or antagonistic correlations between different genera (Zhang et al., 2018). In this section, Pearson’s correlation coefficients explained the microbial relationships among different microbial genera in different regional sausages (Figure 4). *Rahnella*, *Lachnospiraceae AC2044 group*, *Ruminococcaceae UCG 005*, *Unspecified Lachnospiraceae*, *Rikenellaceae RC9 gut group*, and *Ruminococcaceae UCG 010* showed extremely positive patterns, while *Vibrio*, *Myroides*, *Morganella*, *Psychrobacter*, and *Arthrobacter* showed a highly significant co-occurrence pattern. In our study, these microbes of *Vibrio*, *Myroides*, and *Psychrobacter*, regarded as strengthening the aroma profile of meat, showed microbial co-occurrence network results similar to those observed in previous studies (Hu et al., 2020; Wang et al., 2021). Correlation analysis of bacterial genera showed that *Lactobacillus* had weak and negative correlations with almost all other bacterial genera except for *Macrococcus*, *Leuconostoc*, and *Weissella*. Moreover, some LAB with high relative abundance, such as *Lactococcus* and *Lactobacillus*, had no expected and distinct microbial correlation network.

### Volatile compound analysis in the different sausages

A total of 96 volatile compounds were detected by GC–MS in the sausage samples (Table 2) and divided into seven categories, including 19 alcohols, 18 aldehydes, 12 ketones, 10 acids, 11 esters, 3 terpenes, and 23 others. The composition and content of volatile compounds in the different smoked horsemeat sausages varied with region (Supplementary Figure 3). Alcohol is a secondary product of lipid oxidation. Similar to sausages from other regions, the alcohol components detected in the sausages included abundant volatiles (mainly ethanol and 1-hexanol). 1-Octen-3-ol was identified in all samples, which was similar to the results of previous studies (Ansorena et al., 2001; Hu et al., 2020). In addition, some methyl branched alcohols, including 3-methyl-1-butanol, 1-pentanol, and 1-octanol, may have been produced from certain amino acids by LAB and yeast present in the sausages (Sidira et al., 2015). The content and type of 18 aldehydes showed significant differences among samples. The major flavor of processed meat products is usually attributed to compounds with low odor threshold values, such as hexanal, 2-hexenal, and octanal (Zhao et al., 2017). The hexanal content represents the lipid oxidation level.

**TABLE 2:** Content (%) of volatile compounds in smoked horse meat sausages from different regions.

Compounds	C.RI	TC	AL	BL	BZ	YL	ML
**Alcohols**							
Ethanol	938.3	5.328 ± 0.113a	4.297 ± 0.189a	0.547 ± 0.133b	14.473 ± 1.060c	10.516 ± 0.985d	24.398 ± 0.994e
3-methyl-Cyclohexanol	959.2	n.d.	0.470 ± 0.229a	n.d.	n.d.	n.d.	1.647 ± 0.133b
1-Hexadecanol	1095.5	0.982 ± 0.338a	0.208 ± 0.047b	n.d.	1.408 ± 0.205a	3.853 ± 0.627c	n.d.
1-Penten-3-ol	1161.5	0.761 ± 0.388a	0.749 ± 0.157a	0.469 ± 0.208a	0.612 ± 0.252a	n.d.	2.131 ± 0.122b
3-methyl-1-Butanol	1207.8	0.556 ± 0.113a	1.721 ± 0.410	0.341 ± 0.100a	0.480 ± 0.057a	2.270 ± 0.315b	n.d.
1-Pentanol	1246.4	0.998 ± 0.331ab	2.633 ± 0.182c	1.801 ± 0.153bc	0.147 ± 0.047a	17.68 ± 1.235d	3.848 ± 0.260e
2-Penten-1-ol	1305.7	0.519 ± 0.198a	0.489 ± 0.189a	0.198 ± 0.093b	0.054 ± 0.007b	n.d.	0.645 ± 0.075a
1-Hexanol	1352.8	7.860 ± 0.430a	11.507 ± 1.409c	11.169 ± 0.416c	9.089 ± 1.329a	0.051 ± 0.004b	0.918 ± 0.197b
3-Octanol	1392.0	0.031 ± 0.024a	0.080 ± 0.020a	n.d.	1.240 ± 0.153b	n.d.	n.d.
2-Octanol	1417.3	0.122 ± 0.055ab	n.d.	0.211 ± 0.107b	0.178 ± 0.069b	0.066 ± 0.018a	0.199 ± 0.035b
6-Methyl-6-hepten-4-yn-3-ol	1425.3	0.205 ± 0.053ab	n.d.	0.153 ± 0.090a	0.285 ± 0.080b	n.d.	0.187 ± 0.030ab
1-Octen-3-ol	1449.8	1.730 ± 0.331a	7.044 ± 0.367b	2.124 ± 0.289ae	3.564 ± 0.404c	0.108 ± 0.036d	2.493 ± 0.445e
1-Heptanol	1454.5	2.515 ± 0.331a	n.d.	n.d.	0.487 ± 0.157b	n.d.	1.043 ± 0.151c
3-Decen-1-ol	1504.2	2.633 ± 0.224a	0.912 ± 0.139b	3.079 ± 0.351a	5.053 ± 0.265c	0.351 ± 0.153d	8.866 ± 0.352e
Nonenal	1544.1	1.640 ± 0.274a	1.102 ± 0.189b	n.d.	0.909 ± 0.150b	n.d.	1.594 ± 0.156a
1-Octanol	1557.4	1.046 ± 0.252a	1.215 ± 0.241a	1.322 ± 0.265a	1.115 ± 0.150a	0.050 ± 0.004b	0.375 ± 0.149b
2-Octen-1-ol	1616.4	0.067 ± 0.054a	0.319 ± 0.124b	0.250 ± 0.107b	0.237 ± 0.100b	n.d.	0.067 ± 0.011a
Benzeneethanol	1923.4	0.226 ± 0.104a	9.361 ± 0.139b	2.092 ± 0.265c	0.180 ± 0.053a	4.217 ± 0.205d	0.055 ± 0.030a
tetrahydro-2H-Pyran-2-methanol	1973.9	0.059 ± 0.022a	n.d.	0.052 ± 0.018a	n.d.	n.d.	0.427 ± 0.100b
**Aldehydes**							
Hexanal	1082.2	2.925 ± 0.564a	1.382 ± 0.278b	0.868 ± 0.153b	0.252 ± 0.087c	n.d.	3.498 ± 0.309d
(E)-2-Pentenal	1135.6	0.664 ± 0.250a	n.d.	0.322 ± 0.050b	n.d.	n.d.	1.002 ± 0.116c
Heptanal	1187.9	1.954 ± 0.275a	5.794 ± 0.378b	4.224 ± 0.208c	3.031 ± 0.153d	n.d.	2.723 ± 0.265d
2-Hexenal	1224.6	0.701 ± 0.404ac	0.531 ± 0.189a	1.306 ± 0.208b	1.075 ± 0.278c	n.d.	0.669 ± 0.150ac
Octanal	1292.5	2.228 ± 0.393a	6.397 ± 0.273b	4.876 ± 0.200c	3.640 ± 0.252d	0.140 ± 0.042e	1.095 ± 0.252f
2-Heptenal	1330.5	1.556 ± 0.161a	1.491 ± 0.241a	1.507 ± 0.306a	0.227 ± 0.080b	n.d.	2.678 ± 0.265c
Non-anal	1398.2	8.234 ± 0.517a	14.404 ± 1.754b	9.724 ± 0.624a	20.383 ± 0.978c	0.837 ± 0.153d	3.947 ± 0.478e
2,4-Hexadienal	1414.0	0.388 ± 0.250a	n.d.	1.103 ± 0.265b	n.d.	n.d.	1.713 ± 0.451c
(E)-2-Octenal	1437.2	3.792 ± 0.260a	1.905 ± 0.139b	3.632 ± 0.265a	n.d.	0.560 ± 0.097c	3.094 ± 0.265d
3-(methylthio)-Propanal	1465.3	n.d.	0.052 ± 0.010a	0.050 ± 0.033a	n.d.	0.045 ± 0.012a	n.d.
Benzaldehyde	1539.3	1.479 ± 0.292a	1.510 ± 0.139a	2.446 ± 0.153b	0.204 ± 0.101c	0.390 ± 0.153c	1.159 ± 0.289a
2-Decenal	1652.7	1.715 ± 0.180a	1.239 ± 0.241b	2.095 ± 0.252c	0.285 ± 0.076d	n.d.	0.613 ± 0.149e
Benzeneacetaldehyde	1659.0	0.809 ± 0.274a	n.d.	n.d.	0.263 ± 0.088b	0.245 ± 0.067b	0.172 ± 0.060b
(E,E)-2,4-Non-adienal	1713.4	0.474 ± 0.267ac	0.284 ± 0.139ab	0.733 ± 0.361c	n.d.	n.d.	0.232 ± 0.067ab
4-ethyl-Benzaldehyde	1725.1	0.217 ± 0.028a	0.223 ± 0.091a	0.061 ± 0.016b	n.d.	0.186 ± 0.030a	0.164 ± 0.060a
4-Oxohex-2-enal	1778.7	0.170 ± 0.054a	n.d.	0.365 ± 0.265a	n.d.	2.705 ± 0.208b	0.314 ± 0.050a
(E,E)-2,4-Decadienal	1822.8	0.601 ± 0.317a	0.162 ± 0.126c	0.218 ± 0.056bc	n.d.	n.d.	0.470 ± 0.094ab
3,5-dimethyl-Benzaldehyde	1834.2	0.019 ± 0.005a	0.072 ± 0.046ab	0.123 ± 0.050bc	n.d.	n.d.	0.140 ± 0.049c
**Ketones**							
2,3-Pentanedione	1066.4	0.068 ± 0.030ac	n.d.	0.031 ± 0.020b	n.d.	n.d.	0.044 ± 0.012c
3-Heptanone	1155.8	0.370 ± 0.201a	0.319 ± 0.184a	0.058 ± 0.011b	0.025 ± 0.010ab	0.166 ± 0.048b	n.d.
2-Heptanone	1185.2	n.d.	0.770 ± 0.189a	n.d.	0.261 ± 0.072b	8.226 ± 0.231c	n.d.
3-Octanone	1257.6	0.271 ± 0.332ab	0.056 ± 0.026a	n.d.	0.467 ± 0.058b	0.391 ± 0.096b	n.d.
2-Octanone	1286.7	n.d.	0.174 ± 0.069a	n.d.	0.110 ± 0.013a	0.393 ± 0.127b	n.d.
3-hydroxy-2-Butanone	1291.4	3.839 ± 0.317a	n.d.	4.697 ± 0.058b	0.133 ± 0.153c	0.238 ± 0.090c	0.189 ± 0.070c
2,3-Octanedione	1329.7	1.684 ± 0.292a	1.006 ± 0.229b	n.d.	0.170 ± 0.045c	0.238 ± 0.080c	n.d.
6-Octen-2-one	1335.4	0.514 ± 0.194a	0.321 ± 0.094b	n.d.	0.554 ± 0.100a	0.284 ± 0.059b	n.d.
1-Hydroxy-2-butanone	1381.8	0.030 ± 0.004a	n.d.	0.052 ± 0.015a	4.515 ± 0.601b	n.d.	0.191 ± 0.029a
3,5-Octadien-2-one	1528.9	2.027 ± 0.274a	0.662 ± 0.229b	2.024 ± 0.153a	n.d.	n.d.	2.904 ± 0.298c
Dihydro-2(3H)-Furanone	1649.6	0.103 ± 0.092a	0.454 ± 0.319b	0.095 ± 0.024a	0.073 ± 0.042a	0.077 ± 0.019a	0.172 ± 0.048a
Dihydro-5-pentyl-2(3H)-Furanone	2049.0	n.d.	0.095 ± 0.046a	0.023 ± 0.010b	0.044 ± 0.014b	n.d.	n.d.
**Acids**							
Acetic acid	1455.1	2.501 ± 0.361a	6.155 ± 1.718b	5.994 ± 0.252b	4.172 ± 1.009c	0.416 ± 0.095d	0.823 ± 0.118d
Propanoic acid	1541.6	n.d.	n.d.	0.180 ± 0.053a	n.d.	0.081 ± 0.005b	0.216 ± 0.098a
Butanoic acid	1630.2	0.118 ± 0.056a	0.096 ± 0.047a	0.869 ± 0.153b	n.d.	0.066 ± 0.011a	0.112 ± 0.019a
Pentanoic acid	1739.2	n.d.	0.059 ± 0.023a	0.078 ± 0.041a	n.d.	n.d.	0.055 ± 0.009a
Hexanoic acid	1846.6	2.149 ± 0.451a	0.263 ± 0.064b	6.054 ± 0.153c	2.249 ± 0.265ad	2.753 ± 0.451de	3.261 ± 0.179e
Heptanoic acid	1953.3	0.068 ± 0.009a	n.d.	0.055 ± 0.013ab	n.d.	n.d.	0.050 ± 0.017b
Octanoic acid	2060.0	0.064 ± 0.010a	0.134 ± 0.085ab	0.179 ± 0.032b	n.d.	0.165 ± 0.046b	0.165 ± 0.046b
Nonanoic acid	2165.9	0.044 ± 0.013a	0.156 ± 0.047b	0.099 ± 0.025c	0.073 ± 0.023ac	n.d.	0.051 ± 0.024ac
Decanoic acid	2271.8	0.030 ± 0.016a	n.d.	n.d.	n.d.	0.050 ± 0.024a	0.159 ± 0.054b
Benzoic acid	2447.8	0.027 ± 0.014a	n.d.	0.047 ± 0.029a	0.062 ± 0.023a	0.052 ± 0.010a	0.054 ± 0.020a
**Esters**							
Butanoic acid, ethyl ester	1040.5	n.d.	0.264 ± 0.364a	0.024 ± 0.012a	n.d.	n.d.	n.d.
Hexanoic acid, methyl ester	1188.8	0.328 ± 0.132a	n.d.	0.759 ± 0.205b	1.104 ± 0.112c	n.d.	2.843 ± 0.279d
Hexanoic acid, ethyl ester	1236.3	1.225 ± 0.101a	0.465 ± 0.139bc	0.636 ± 0.204b	n.d.	0.218 ± 0.100c	2.707 ± 0.259d
Acetic acid, hexyl ester	1275.5	0.040 ± 0.008a	0.078 ± 0.073a	0.05 ± 0.014a	n.d.	21.166 ± 1.453b	n.d.
Heptanoic acid, ethyl ester	1337.3	0.596 ± 0.246a	n.d.	n.d.	n.d.	n.d.	0.101 ± 0.018b
Octanoic acid, methyl ester	1392.6	0.289 ± 0.194a	n.d.	0.052 ± 0.013b	n.d.	1.098 ± 0.173c	0.423 ± 0.070a
Octanoic acid, ethyl ester	1442.8	n.d.	n.d.	n.d.	0.224 ± 0.129a	n.d.	3.230 ± 0.344b
Decanoic acid, methyl ester	1598.7	0.101 ± 0.019a	n.d.	0.036 ± 0.017a	n.d.	0.230 ± 0.085b	0.401 ± 0.107c
Decanoic acid, ethyl ester	1641.8	0.399 ± 0.202a	n.d.	n.d.	n.d.	0.218 ± 0.03b	0.468 ± 0.076a
n-Caproic acid vinyl ester	1670.6	0.746 ± 0.205a	0.375 ± 0.139b	2.004 ± 0.153c	0.815 ± 0.306a	0.218 ± 0.095b	0.546 ± 0.087ab
Dodecanoic acid, methyl ester	1805.7	0.294 ± 0.175a	n.d.	n.d.	n.d.	0.008 ± 0.004b	0.024 ± 0.007b
**Terpenes**							
D-Limonene	1194.1	n.d.	0.084 ± 0.008a	0.952 ± 0.105ab	1.388 ± 0.205b	6.432 ± 1.358c	n.d.
2-Undecenal	1761.4	0.551 ± 0.161a	0.978 ± 0.091b	0.561 ± 0.20a	0.226 ± 0.100c	0.105 ± 0.009c	0.155 ± 0.043c
tetradecyl-Oxirane	2140.0	0.055 ± 0.018a	0.396 ± 0.182b	n.d.	0.346 ± 0.166b	0.063 ± 0.023a	n.d.
**Others**							
2,7,10-trimethyl-Dodecane	1039.2	0.088 ± 0.038a	0.75 ± 0.391b	n.d.	0.257 ± 0.151a	0.170 ± 0.059a	n.d.
4-ethyl-Heptane	1052.6	n.d.	0.268 ± 0.091a	n.d.	0.220 ± 0.119a	0.175 ± 0.050a	n.d.
4-methyl-Decane	1056.0	n.d.	0.508 ± 0.189a	0.172 ± 0.053b	0.202 ± 0.083b	0.197 ± 0.032b	n.d.
Ethyl-benzene	1125.7	n.d.	0.279 ± 0.168a	0.192 ± 0.076a	n.d.	n.d.	n.d.
1,4-dimethyl-Benzene	1130.8	n.d.	0.397 ± 0.132a	1.147 ± 0.096b	0.264 ± 0.054c	0.199 ± 0.057c	n.d.
p-Xylene	1141.2	n.d.	0.291 ± 0.193a	1.683 ± 0.153b	n.d.	n.d.	n.d.
Pentadecane	1169.6	n.d.	0.249 ± 0.136a	n.d.	0.190 ± 0.045a	n.d.	n.d.
Heptadecane	1173.6	n.d.	0.208 ± 0.137	n.d.	n.d.	n.d.	n.d.
o-Xylene	1185.6	n.d.	1.339 ± 0.262a	0.705 ± 0.282b	n.d.	n.d.	n.d.
3,6-dimethyl-Undecane	1197.1	0.383 ± 0.205a	1.016 ± 0.278b	n.d.	n.d.	n.d.	n.d.
4-methyl-Dodecane	1203.9	0.037 ± 0.017a	n.d.	n.d.	0.071 ± 0.014a	n.d.	0.144 ± 0.062b
4,6-dimethyl-Dodecane	1232.3	n.d.	0.209 ± 0.126a	n.d.	0.443 ± 0.150b	0.166 ± 0.054a	n.d.
2-pentyl-Furan	1233.7	0.393 ± 0.165ab	0.662 ± 0.364bc	0.730 ± 0.071c	n.d.	0.112 ± 0.012ad	0.204 ± 0.051ad
2,4,6-trimethyl-Decane	1243.1	n.d.	n.d.	0.522 ± 0.208a	0.680 ± 0.252a	n.d.	n.d.
Styrene	1263.2	0.765 ± 0.260a	1.401 ± 0.213b	7.934 ± 0.348c	0.089 ± 0.033d	1.214 ± 0.264b	0.334 ± 0.071d
Hexadecane	1297.6	0.161 ± 0.048a	0.752 ± 0.197b	n.d.	0.486 ± 0.113c	n.d.	0.077 ± 0.012a
2,4-Heptadiena	1475.1	1.105 ± 0.319a	0.204 ± 0.043b	1.122 ± 0.252a	n.d.	0.073 ± 0.061b	6.675 ± 0.568c
Diallyl disulfide	1489.0	20.999 ± 2.153a	0.328 ± 0.139b	1.497 ± 0.219b	9.003 ± 0.314c	0.053 ± 0.019b	n.d.
4-bromo-Octane	1548.7	n.d.	n.d.	1.154 ± 0.145a	n.d.	n.d.	0.442 ± 0.078a
2-methoxy-Phenol	1873.9	0.675 ± 0.222a	n.d.	n.d.	1.501 ± 0.316b	3.138 ± 0.404c	n.d.
2-methyl-Phenol	2011.6	0.656 ± 0.274a	0.145 ± 0.070b	0.036 ± 0.010b	0.068 ± 0.013b	2.373 ± 0.355c	0.149 ± 0.172b
Phenol	2015.6	0.867 ± 0.164a	0.079 ± 0.056a	0.035 ± 0.017a	0.026 ± 0.009a	4.019 ± 1.061b	0.058 ± 0.058a
2,3-dimethyl-Phenol	2185.0	0.140 ± 0.111a	n.d.	n.d.	0.619 ± 0.201b	0.491 ± 0.153b	n.d.

Mean value ± SE (n = 3) followed by different lower-case letters in each column indicate significant differences at P < 0.05.

A total of 12 ketones were detected in all samples. Methyl ketones, such as 3-heptanone and 2-octanone, which were derived from incomplete microbial β-oxidation due to the presence of *Carnobacterium* and *Psychrobacter*, accounted for a higher proportion (Laursen et al., 2006; Irlinger et al., 2012). In addition, some ketones provide products with a characteristic fermented flavor, possibly via amino acid catabolism mediated by LAB (Kieronczyk et al., 2004). Medium-chain (C6–C12) and short-chain (C < 6) acids were identified in all samples, greatly affecting flavor development due to their lower threshold values (Sidira et al., 2015).

The concentration and type of acids detected in this experiment were significantly discrepant, which might be due to different intrinsic and extrinsic factors. The esterification of short-chain acids with alcohols generally leads to the formation of esters. Esters were generally detected at high levels, which might have been due to the existence of precursors, such as ethanol and decanoic acid (Curioni and Bosset, 2002). These esters provided a fruity and floral flavor and masked the rancid odor of smoked horsemeat sausage. In addition to the above, the levels of terpenes and other long chain alkanes were significantly variable among the samples (*P* < 0.05) due to the differences among sausage manufacturers and traditional techniques.

**FIGURE 5:**
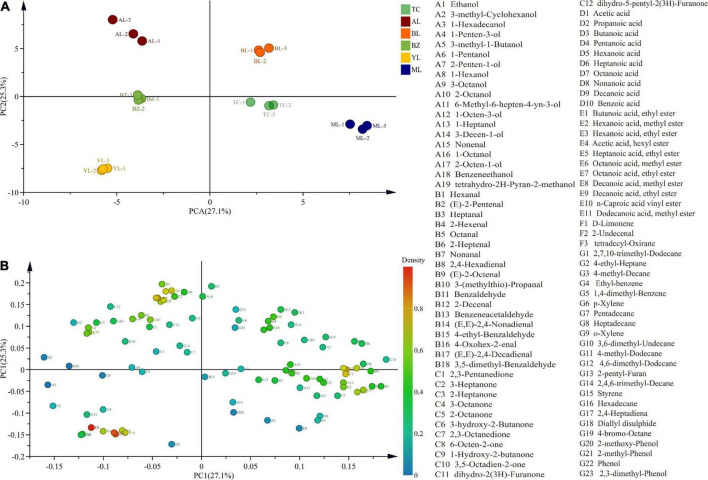
Principal component analysis (PCA) score plot **(A)** and loading plot **(B)** of volatile compounds from horsemeat sausages from different regions.

Principal component analysis was applied to establish a model to indicate the differences in aroma components of smoked horse meat from different regions. The R2X and Q2 values of the obtained model were both greater than 0.5 (Supplementary Figure 4), indicating that the established model could be used to explain the differences in aroma components between samples. The PCA results showed that PC1 and PC2 explained 27.1 and 25.3% of the variance, respectively. In the score plot (Figure 5A), the smoked horsemeat sausage samples from the same region were clustered into one class, and the smoked horsemeat sausage samples from different regions could be divided into four classes, where the BZ and YL were clustered into one class, TC and ML samples were clustered into one class, and AL and BL samples were clustered into one class, which was roughly the same as the result of PCA of the bacterial community. In the loading plot (Figure 5B), 1-penten-3-ol, 2-octen-1-ol, 2-heptanone, d-limonene, 2-methyl-phenol, and phenol contributed the most to the separation of different samples.

### Screening of core microorganisms and volatile components

**FIGURE 6:**
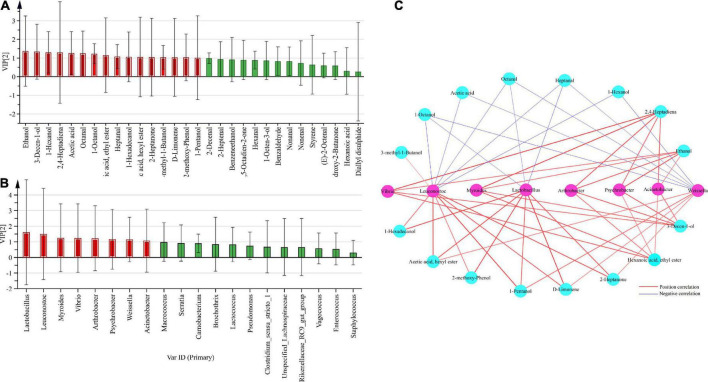
Analysis of the core microbes and volatile compounds by O2PLS modeling. **(A)** VIP (variable importance for predictive components) plot of bacteria vs. the top 30 volatile compounds. **(B)** VIP plot of volatile compounds vs. the top 20 bacteria. **(C)** Correlation analyses of core bacteria and volatile compounds.

The top 30 volatile components in terms of relative abundance in smoked horsemeat sausage samples from different regions were selected to identify the core volatile components. VIP analysis was used to identify volatile components closely related to bacterial groups, where volatile components were set as variables (x), bacteria were set as attribute variables (y), and volatile components with VIP values > 1 were identified as core volatiles. The constituents are marked in red (Figure 6A) and are defined as the dominant core volatile constituents in smoked horsemeat sausage. The results showed that 11 components, including octanal, 1-octanol, heptanal, and 1-hexadecanol, were identified as core volatile components. Using the same method, the top 20 bacteria (genus level) in terms of relative abundance in smoked horsemeat sausage samples from different regions were selected to identify core bacteria, and VIP analysis was used to identify bacterial groups closely related to volatile compounds, where bacterial groups were set as variables (x), volatile compounds were set as attribute variables (y), and bacterial groups with VIP values > 1 were identified as core bacterial groups and are marked in red (Figure 6B), indicating that these core bacterial groups play an important role in the formation of volatile components. The results showed that *Leuconostoc*, *Myroides*, *Arthrobacter*, *Psychrobacter*, *Weissella*, *Acinetobacter*, and *Lactobacillus* were identified as core bacterial groups, being also present in various types of dry sausages.

### Correlation analysis between bacteria and flavors

Although the raw materials and process conditions resulted in variation in the volatile compounds in different dry sausage samples, the bacterial community was also a key factor affecting the volatile flavor development of smoked horsemeat sausage. As previously reported, the quality of fermented meat is highly dependent on the indigenous bacterial metabolic activities and fermentation behaviors (Ammor and Mayo, 2007; Zhang et al., 2019). In our work, the Spearman correlation coefficients between core bacteria and the core volatile compounds were calculated using O2PLS (Supplementary Table 1). As shown in Figure 6C, *Vibrio*, *Myroides*, *Arthrobacter*, *Psychrobacter*, *Acinetobacter*, and *Weissella* had positive correlations with the four core volatile compounds. There were positive correlations between the levels of *Leuconostoc* and *Lactobacillus* and those of 1-hexadecanol, hexyl acetate, 2-methyl-phenol, 1-pentanol, d-limonene, and 2-heptanone. *Lactobacillus*, *Weissella*, and *Leuconostoc* are probiotics that have beneficial effects on human health and are widely used as starters in many fermentation processes (Leroy et al., 2006; Galle et al., 2010). In addition, there were negative correlations between the levels of *Leuconostoc*, *Lactobacillus*, and *Weissella* and those of 1-octanol, acetic acid, octanal, heptanal, and 1-hexanol.

Microorganisms can produce some extracellular enzymes, such as proteases, lipidase, and esterases (Woo et al., 2014), which can make full use of the precursors in meat to form more volatile compounds. *Lactobacillus*, *Vagococcus*, *Staphylococcus*, and *Carnobacterium* were the main sources of esters, especially decanoic acid ester. These microbes that increase the levels of organic acids, short-chain fatty acids, and esters during meat fermentation were widely present in the different sausage samples (Cruxen et al., 2017; Hu et al., 2020), which was consistent with our results. Overall, these microbes, with some potentially important enzymatic characteristics, can affect the matrix volatile metabolism.

## Conclusion

We conducted a field experiment to investigate the differences in physicochemical properties, bacterial communities, and volatile compounds in smoked horsemeat sausages collected from different regions in Xinjiang, China. Our study revealed that the abundance of *Lactobacillus* and *Vagococcus* was significantly higher in the TC samples than in the BZ and BL samples, whereas that of *Lactococcus* and *Carnobacterium* was markedly higher in the BL samples than in the other samples. In the functional analyses, most of the genes were classified into the metabolism function group, in which genes involved in carbohydrate metabolism had the highest abundance. The pathways involved in the production of lactic acid in carbohydrate metabolism were C6, C8, and C14. In addition, alcohols, aldehydes and esters were the three dominant groups of volatile compounds, in addition to ketones, especially in the AL and BL samples. There was a significant correlation between bacterial communities and volatile compounds in smoked horsemeat sausages. Further research should be devoted to determination of the bacterial functions and fermentation mechanisms in smoked horsemeat sausages by using a multiomics approach, such as metagenomics, metaproteomics, and metatranscriptomics. This study provided valuable information about the microbial composition and diversity in smoked horsemeat sausages, which will help improve the uniformity and palatability of Xinjiang smoked horsemeat sausages and maintain quality consistency.

## Data availability statement

The datasets presented in this study can be found in online repositories. The names of the repository/repositories and accession number(s) can be found below: https://www.ncbi.nlm.nih.gov/, PRJNA837620.

## Author contributions

LJ did the experiment, collected the test data, and drafted the manuscript. YC and FL contributed the data curation and software. LD contributed the data curation. TW analyzed data. XS conceived and designed the study. BW revised the manuscript. All authors contributed to the article and approved the submitted version.

## Conflict of interest

The authors declare that the research was conducted in the absence of any commercial or financial relationships that could be construed as a potential conflict of interest.

## Publisher’s note

All claims expressed in this article are solely those of the authors and do not necessarily represent those of their affiliated organizations, or those of the publisher, the editors and the reviewers. Any product that may be evaluated in this article, or claim that may be made by its manufacturer, is not guaranteed or endorsed by the publisher.
