# Supplementary material for: Bacterial community diversity and its potential contributions to the flavor components of traditional smoked horsemeat sausage in Xinjiang, China
Source: Front Microbiol. 2022 Jul 27;13:942932. doi: 10.3389/fmicb.2022.942932 (PMC9365192; doi:10.3389/fmicb.2022.942932)
Supplement: Supplementary file 1 [file Table_1.DOC]

**Supplementary Information for**

**Bacterial community diversity and its potential contributions to the flavour components of traditional smoked horsemeat sausage in Xinjiang, China**

Lei Jiang^a^, Yu Chen^b, c^, Li Deng^b^, Fei Liu^a^, Tengbin Wang^d^, Xuewei Shi^b*^, Bin Wang^b*^

1. College of Life and Geographical Sciences, Kashi University, Kashi 844000, China
2. Food College, Shihezi University, Shihezi 832000, China
3. College of Enology, Northwest A&F University, Yangling, Shanxi, 712100, China
4. Xinjiang Academy of Analysis and Testing, Wulumuqi 830000, China

*Corresponding authors

E-mail addresses: B. W.: [binwang0228@shzu.edu.cn](mailto:binwang0228@shzu.edu.cn); X. S.: shixuewei@shzu.edu.cn

Tel.: +86-0993-2058093

*Bin Wang and Xuewei Shi contributed equally to this work. Author order was determined by drawing straws.Supplementary tables:

**Table S1.** Richness and diversity indexes of bacteria communities on the surface of horse meat sausages.

| Sample | Reads | Observed OTUs | Ace | Chao1 | Good's coverage |
| --- | --- | --- | --- | --- | --- |
| TC | 54749 | 107 | 107.00 | 107.00 | 1 |
| AL | 51842 | 33 | 33.00 | 33.00 | 1 |
| BL | 50861 | 44 | 44.00 | 44.00 | 1 |
| BZ | 69159 | 140 | 140.00 | 140.00 | 1 |
| YL | 57982 | 113 | 113.469311 | 113.50 | 0.9999 |
| ML | 46503 | 187 | 187.20 | 187.00 | 0.9999 |

## **Table S2.** The linear correlation coefficients between eight selected bacteria and volatile compounds.

| **Volatile compounds** | *Lactobacillus* | *Leuconostoc* | *Myroides* | *Vibrio* | *Arthrobacter* | *Psychrobacter* | *Weissella* | *Acinetobacter* |
| --- | --- | --- | --- | --- | --- | --- | --- | --- |
| Ethanol | 0.0562622 | 0.299155 | 0.822252 | 0.822527 | 0.907078 | 0.947923 | 0.398706 | 0.730098 |
| 3-Decen-1-ol | -0.391715 | -0.230617 | 0.845282 | 0.840464 | 0.901519 | 0.915117 | 0.0716234 | 0.829609 |
| 1-Hexanol | -0.58966 | -0.832675 | -0.568311 | -0.58713 | -0.567088 | -0.552426 | -0.843785 | -0.537401 |
| 2,4-Heptadiena | -0.349957 | 0.0206314 | 0.980775 | 0.978705 | 0.934255 | 0.86671 | 0.32839 | 0.88121 |
| Acetic acid | -0.556477 | -0.725813 | -0.494282 | -0.505653 | -0.501949 | -0.494717 | -0.688121 | -0.408762 |
| Octanal | -0.642041 | -0.73149 | -0.410169 | -0.427452 | -0.424679 | -0.424754 | -0.7214 | -0.404693 |
| 1-Octanol | -0.671185 | -0.914369 | -0.456307 | -0.477108 | -0.452054 | -0.439855 | -0.872145 | -0.430889 |
| Hexanoic acid, ethyl ester | -0.427038 | -0.0581404 | 0.905261 | 0.895822 | 0.824461 | 0.731969 | 0.205736 | 0.727179 |
| Heptanal | -0.817491 | -0.749896 | -0.059587 | -0.0799332 | -0.0908058 | -0.114705 | -0.643519 | -0.111986 |
| 1-Hexadecanol | 0.95527 | 0.823147 | -0.355126 | -0.333128 | -0.29899 | -0.239823 | 0.547178 | -0.274324 |
| Acetic acid, hexyl ester | 0.935744 | 0.950693 | -0.199099 | -0.169697 | -0.203844 | -0.194861 | 0.764952 | -0.0938349 |
| 2-Heptanone | 0.916186 | 0.939366 | -0.227489 | -0.199703 | -0.230532 | -0.218989 | 0.731216 | -0.137537 |
| 3-methyl-1-Butanol | 0.57778 | 0.618223 | -0.493011 | -0.483597 | -0.520721 | -0.519684 | 0.287705 | -0.539145 |
| D-Limonene | 0.976906 | 0.899728 | -0.285897 | -0.252662 | -0.255351 | -0.216939 | 0.729892 | -0.100026 |
| 2-methoxy-Phenol | 0.956191 | 0.787392 | -0.344307 | -0.321444 | -0.261699 | -0.182865 | 0.527197 | -0.232843 |
| 1-Pentanol | 0.865638 | 0.978855 | -0.047195 | -0.0179044 | -0.0750805 | -0.0881305 | 0.836903 | 0.0315857 |

# Supplementary figures


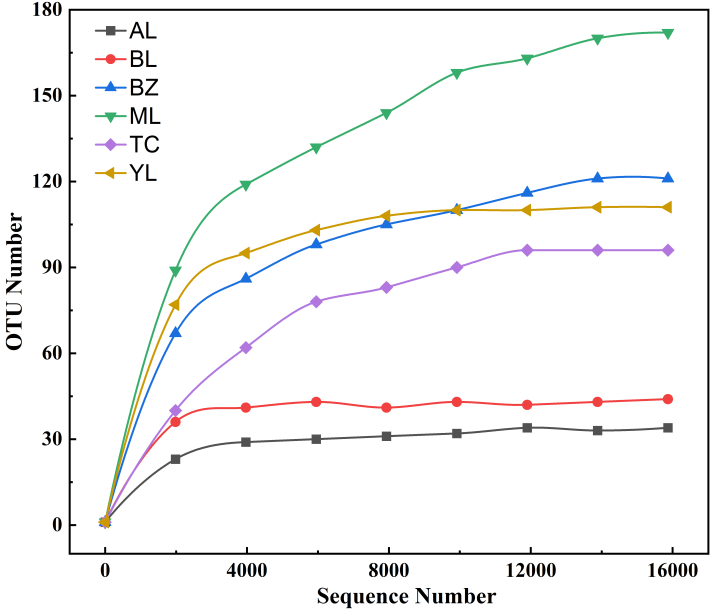


## **Figure S1.** Richness rarefaction curves of Xinjiang traditional smoked horsemeat sausage from different regions based on the OTUs of bacteria.


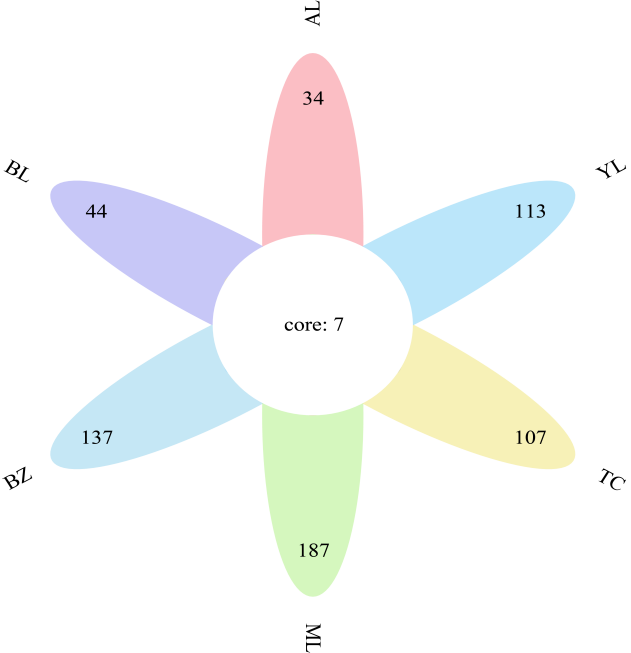


## **Figure S2.** Petal diagrams of Xinjiang traditional smoked horsemeat sausage from different regions based on the OTUs of bacteria.


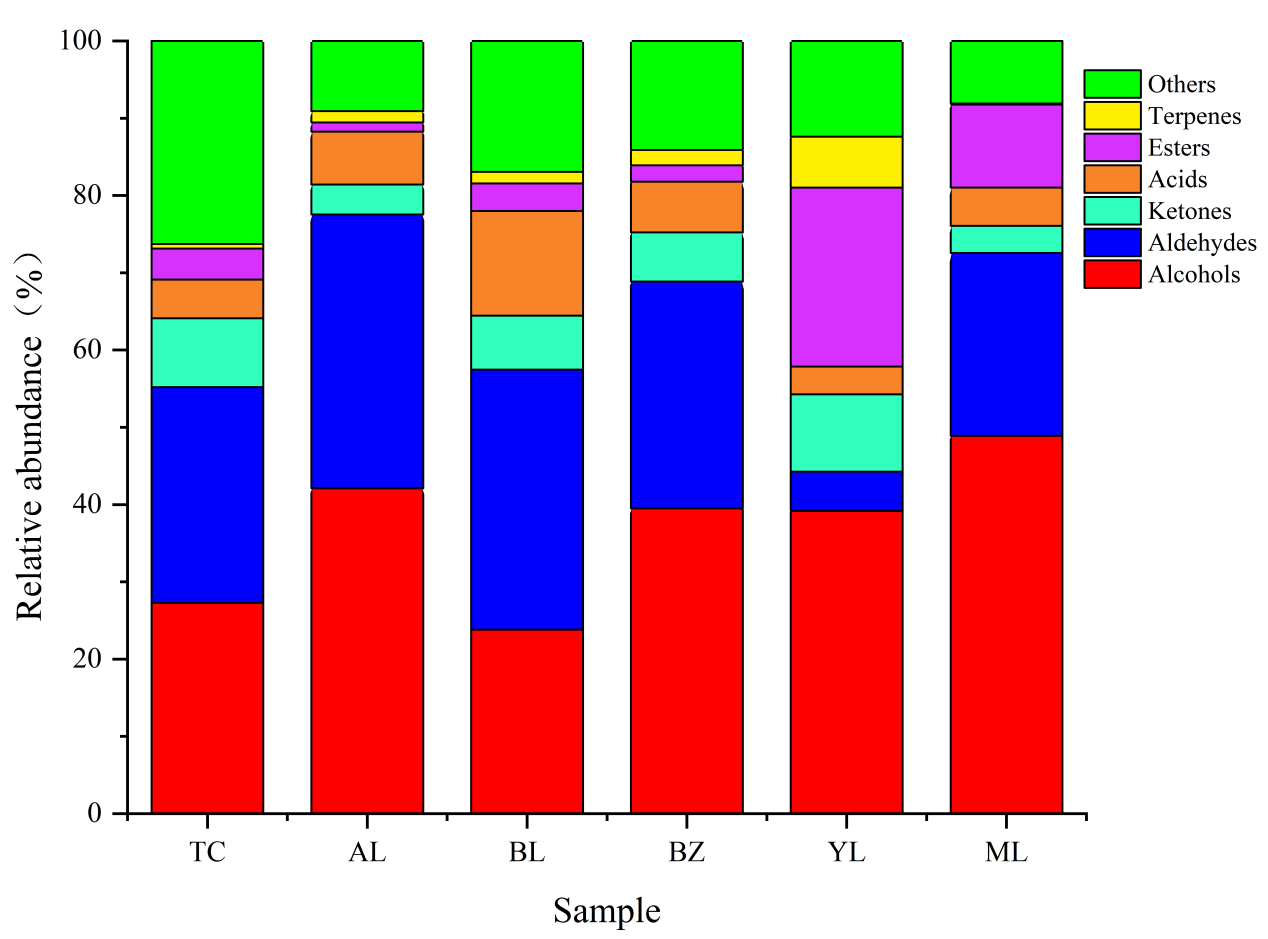


**Figure S3**. The composition of volatile compounds of Xinjiang smoked horse meat sausage from different regions.


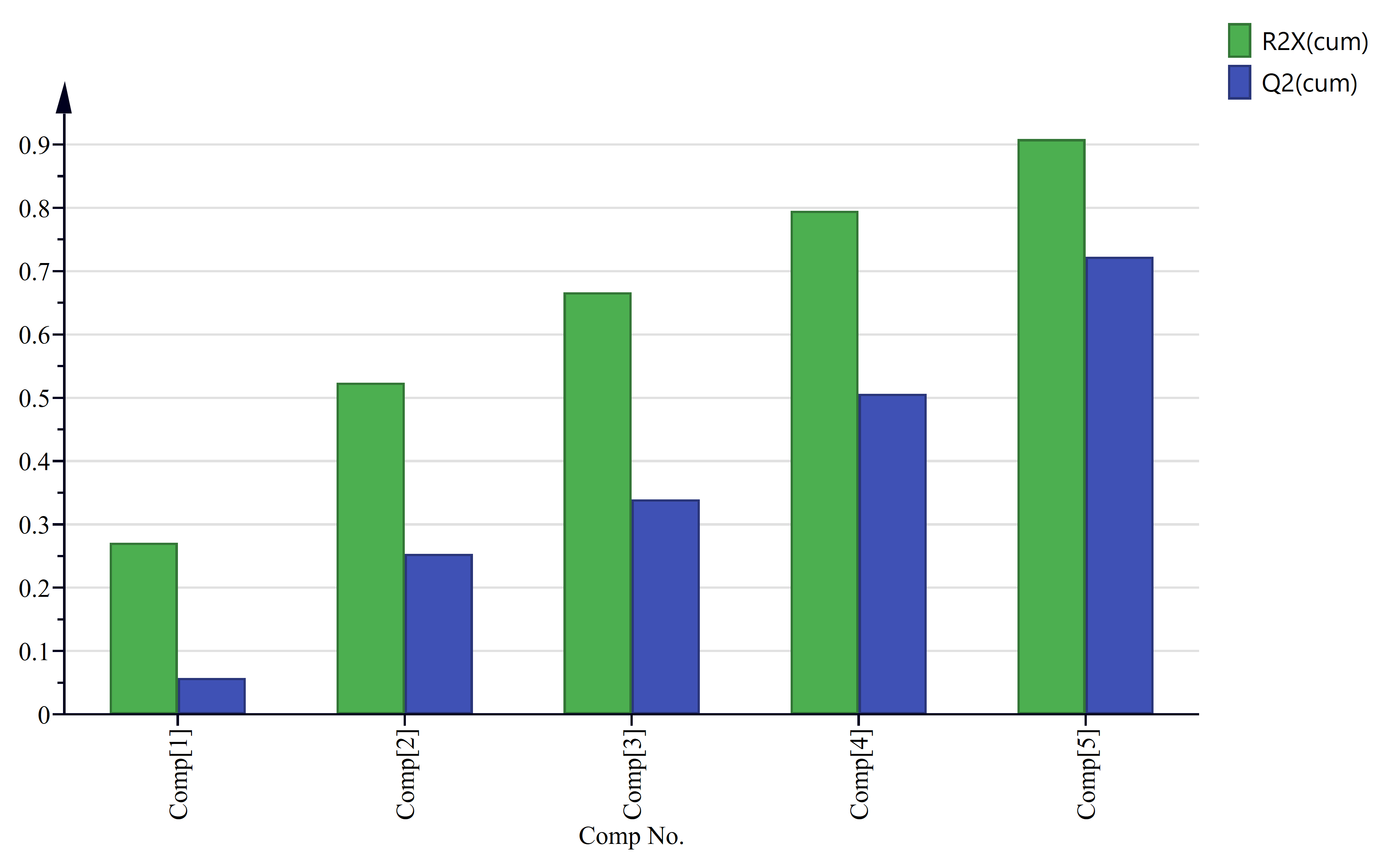


## **Figure S4.** Quality assessment model of volatile compounds
